# Covalent Grafting Terbium Complex to Alginate Hydrogels and Their Application in Fe^3+^ and pH Sensing

**DOI:** 10.1002/gch2.201800067

**Published:** 2018-11-13

**Authors:** Zeyu Zhang, Fengyi Liu, Quanqing Xu, Han Zhu, Aixin Zhu, Junfeng Kou

**Affiliations:** ^1^ College of Chemistry and Chemical Engineering Yunnan Normal University Kunming 650500 China

**Keywords:** alginate, covalent‐grafting, hydrogels, luminescence, sensors

## Abstract

Biocompatible luminescent hydrogels containing covalently linked terbium complexes with a macrocyclic ligand are prepared by a facile method. The environmentally friendly preparation procedure is carried out at room temperature using water as a solvent. These new hybrid materials can act as luminescent sensors to detect Fe^3+^ with relative selectivity and high sensitivity. The hydrogels also show pH sensing with a wide range.

In recent years, polysaccharides‐based hydrogels have drawn considerable attention since they can be used in numerous applications, from food and cosmetic processing to tissue engineering and drug delivery.^1^, ^2^, ^3^, ^4^ Specifically, alginate hydrogel has been studied extensively in the field of biomedical field as scaffold materials. Alginate (Alg), a polysaccharide of seaweed origin, is extracted from brown marine algae.^5^ Thanks to its excellent biocompatibility and low toxicity, alginate is widely used in the food and beverage industry as a thickening, gelling, or colloidal agent.^6^, ^7^, ^8^

Typically, alginates form heat‐stable gels with metal cations, such as Ca^2+^ and the first transition metal (Cr^3+^, Mn^2+^, Fe^3+^, Co^2+^, Ni^2+^, Cu^2+^, Zn^2+^, etc.).^9^, ^10^ It is also noted that alginates form gels with lanthanide ions to give photoluminescent hybrid materials.^11^ For about two decades, there has been a strong interest in lanthanide hybrid materials due to their intriguing optical properties (such as narrow emission bands, large Stoke shifts, and long luminescence lifetimes).^12^, ^13^, ^14^ The design of new luminescent hybrid materials based on covalently bonded lanthanide complexes has been a very active research field. The advantages of covalent attachment of lanthanide complexes to the silica matrix are higher doping concentrations, better homogeneity, and the utilization of the so‐called antenna effect (or sensitization) via the polydentate ligands. However, it should be noted that some complex synthetic and purification protocols are typically employed in order to obtain hydrolyzable trialkoxysilyl derivatives of polydentate ligands that can coordinate to the lanthanide ion. Hence, an environmentally friendly preparation approach is much needed under this view.

So far most of the studies of lanthanide hybrid materials were concentrated on silica‐based system.^15^, ^16^ To the best of our knowledge, no studies are available on the covalently grafting photoluminescent lanthanide complexes to polysaccharides‐based matrix. Compared to polysaccharides‐based materials, silica‐based hybrid materials show poorer biocompatibility and biodegradability, which greatly limited them for biological applications such as bioimaging and biolabeling. Another challenge is to prepare luminescent hydrogels that are sufficiently bright in aqueous environment, because the vibrational states of high‐frequency OH oscillators of water ligand can greatly decrease the luminescence efficiency of the lanthanide ions.

It is also known that luminescent sensing of Fe^3+^ ions is of practical significance, because many vital cell functions of either human or other animals depend closely on the specific amount of Fe^3+^ involved, which can not only facilitate the formation of hemoglobin and muscle but also improve the brain functions.^17^, ^18^, ^19^ Although there are some reports about the lanthanide luminescence application in Fe^3+^ sensing, as far as we know, no reports have been reported using polysaccharides‐based hydrogel hybrid materials as sensors to detect Fe^3+^.

Herein, we wish to report a facile method to prepare highly luminescent hydrogels and their applications to detect Fe^3+^ and as pH sensors. The preparation was carried out at room temperature using water as solvent. The lanthanide complexes with macrocyclic ligand TCPP (TCPP = tetrakis(4‐carboxyphenyl)porphyrin) have been covalently grafted to the alginate network via ligand replacement.

The luminescent hydrogels with terbium complex were prepared by a two‐step method. The first step of preparation is similar to our previously published paper.^11^ The Tb^3+^‐Alg hydrogels were obtained by adding the alginates solution to an aqueous solution of TbCl_3_ under stirring at room temperature using a syringe with a 0.8 mm diameter needle. After washing the Tb^3+^‐Alg hydrogels with distilled water sufficiently, these hydrogels were then added to the solution of TCPP which was adjusted by dilute NaOH solution to pH 10. The mixture was kept for 12 h under mild stirring. Then the hydrogels (designated as Tb^3+^‐Alg‐TCPP) were separated and washed copiously with distilled water. In order to investigate the inner textural properties of the Tb^3+^‐Alg‐TCPP hydrogels, the corresponding aerogels materials were also prepared using the supercritical CO_2_ drying technique.

The typical SEM images of the Tb^3+^‐Alg‐TCPP aerogel samples show the porous structures of the hybrid materials (**Figure** **1**). These macropores and mesopores were made of randomly aligned alginate fibrils.

**Figure 1:**
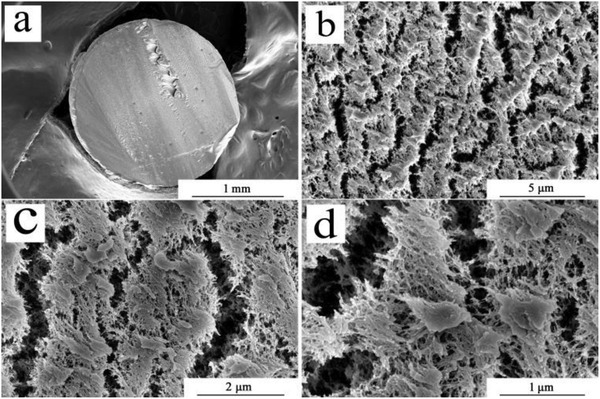
The cross‐sectional micrographs of Tb^3+^‐Alg‐TCPP aerogels with a magnification of a) 0.10 kx, b) 20.00 kx, c) 50.00 kx, d) 100.00 kx.

**Figure** **2** depicts the mapping of the Tb, O, and N elements, showing that these three elements were dispersed uniformly throughout the alginate matrix, and there were no agglomerations observed since the covalently grafting of terbium complexes to the matrix were carried out at molecular level.

**Figure 2:**
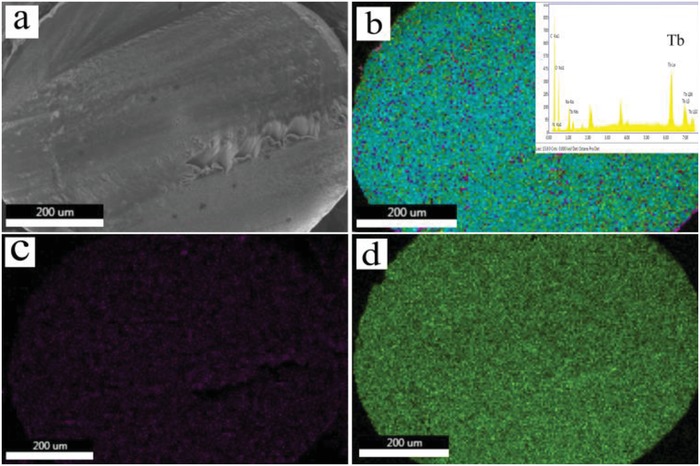
a) SEM of cross section of Tb^3+^‐Alg‐TCPP aerogels. b) X‐ray mapping of the relative Tb (green), O (yellow), and N (purple) content, respectively. c) N and d) Tb distributions. Inset: the EDX‐analysis for the Tb^3+^‐Alg‐TCPP aerogels.

In order to analyze the specific surface area and the diameter of porous structure, the nitrogen adsorption–desorption isotherms were measured at 77 K (Figure S1, Supporting Information). According to the IUPAC classification,^20^ the obtained isotherms can be classified as type ІV which indicates that the obtained aerogels showed mesoporous structure with strong adsorbate–adsorbent interaction.^21^, ^22^ There is a hysteresis loop caused by the capillary condensation in the mesoporous (2–50 nm) at higher pressure. Usually, large surface area is beneficial for practical application such as catalysis, sensing, etc. The specific surface areas of aerogel samples were defined from the adsorption–desorption isotherms. The Brunauer–Emmett–Teller (BET) surface area of Tb^3+^‐Alg‐TCPP aerogels is 517.982 m^2^ g^−1^. The size distributions of pore diameter were determined from desorption isotherms by the Barret–Joyner–Halenda (BJH) method.^23^ The porosity data indicates that the total pore volume of Tb^3+^‐Alg‐TCPP aerogels is 4.754 cm^3^ g^−1^. The higher‐magnification SEM images and the pore size distribution analysis showed that Tb^3+^‐Alg‐TCPP aerogels are porous material with macropore–mesopore structure.

The photoluminescence properties of the Tb^3+^‐Alg‐TCPP hydrogels were characterized by excitation and emission spectra (**Figure** **3**d, black line). The excitation at 233 nm leads to four sharp emission peaking located at 489, 544, 584, and 622 nm, ascribed to the ^5^D_4_→^7^F_J_ (J = 6–3) transitions, of which the emission at 544 nm is the most prominent one, which is responsible for the green emission.^24^, ^25^ It is noted that after the covalent grafting of TCPP, the Tb^3+^‐Alg‐TCPP hydrogels showed obvious luminescence enhancement (Figure S2, Supporting Information), which may be due to the ligand replacement of H_2_O by TCPP. Through the coordination with carboxylic group of TCPP, the so called “antenna effect” was realized, leading to the effective energy transfer from the ligand TCPP to Tb^3+^ (Figure S3, Supporting Information).

**Figure 3:**
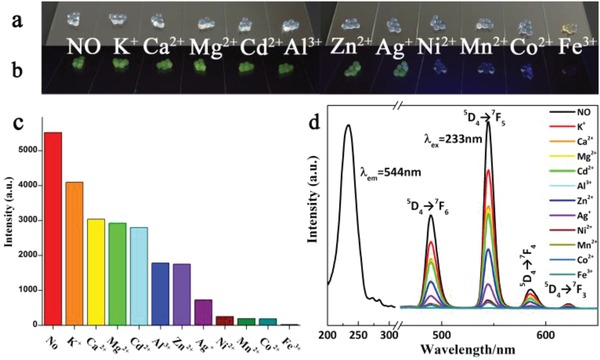
Fluorescence images of the Tb^3+^‐Alg‐TCPP aerogels after immersion into solutions with different metal ions (1 × 10^−3^ m) a) exposed to daylight and b) under the irradiation of UV light. c,d) Photoluminescence intensity of the ^5^D_4_→^7^F_5_ transition (λ_em_ = 544 nm) of Tb^3+^‐Alg‐TCPP aerogels treated with different metal ions (1 × 10^−3^ m). “NO” denotes the fluorescence intensity of Tb^3+^‐Alg‐TCPP hydrogels without metal ions.

In the past decades, fluorescent sensors as a simple, sensitive, and economical method for online monitoring have been widely used as the metal cations selective detectors.^26^, ^27^ Herein, we investigated the Tb^3+^‐Alg‐TCPP hydrogels as fluorescent sensors for Fe^3+^ ions. The hybrid materials with porous structures show high surface area, which is beneficial for the sensing of Fe^3+^. To examine and compare the sensing potential of the obtained hybrid materials, the Tb^3+^‐Alg‐TCPP hydrogels were first suspended in solutions containing different metal ions (K^+^, Ca^2+^, Mg^2+^, Cd^2+^, Al^3+^, Zn^2+^, Ag^+^, Ni^2+^, Mn^2+^, Co^2+^, and Fe^3+^). Most metal ions have varying degrees of quenching effects on luminescence intensity and Fe^3+^ revealed significant quenching effect on the luminescence for Tb^3+^‐Alg‐TCPP (Figure 3).

In order to further investigate the quenching effect of Fe^3+^, luminescence quenching with mixture of metal ions has been performed. It is found that without Fe^3+^, the mixture of metal ions showed only partial quenching. The quenching is almost complete when Fe^3+^ is included, which evidenced that quenching is mainly due to Fe^3+^ ions (Figure S4, Supporting Information).

To further study of the quenching effect of Tb^3+^‐Alg‐TCPP hydrogels with Fe^3+^ ions, luminescence titration was conducted with the addition of different concentrations of Fe(NO_3_)_3_ to Tb^3+^‐Alg‐TCPP hydrogels. It was observed that Tb^3+^‐Alg‐TCPP hydrogels exhibited obvious responses with increasing the concentration of Fe^3+^ ions (**Figure** **4**a,b). The luminescence quenching data were calculated according to the Stern–Volmer equation as follows^28^:

$$\left(I_{0}/I\right)\;-\;1\;=K_{\mathrm{sv}}\;\times \;\left[M\right]$$

where *I*_0_ and *I* are the luminescent intensity before and after Fe^3+^ ions treatment, respectively; [m] is the Fe^3+^ ions molar concentration; and *K*_sv_ is the Stern–Volmer constant. Inset of Figure 4a shows the *K*_sv_ curve of Tb^3+^‐Alg‐TCPP with Fe^3+^ ions. The linear correlation coefficient (*R*) is 0.99448, which suggests that the quenching effect of Fe^3+^ on the luminescence of Tb^3+^‐Alg‐TCPP shows good accordance with the Stern–Volmer model. The *K*_sv_ value is calculated to be 2.73 × 10^5^ L mol^−1^, which is about two orders of magnitude higher than the previously published results.^29^, ^30^ The high *K*_sv_ value reveals a strong quenching effect on the luminescence of Tb^3+^‐Alg‐TCPP hydrogels. The experiment result indicates that Tb^3+^‐Alg‐TCPP hydrogels may be used as a fluorescence sensor for detecting Fe^3+^ ions with high sensitivity and selectivity.

**Figure 4:**
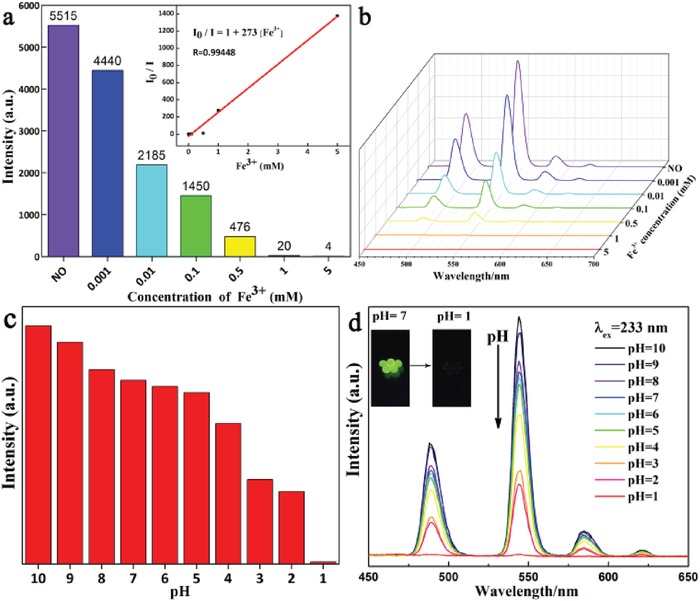
a,b) Fluorescence intensity (λ_em_ = 544 nm) of Tb^3+^‐Alg‐TCPP hydrogels under different concentrations of Fe^3+^ aqueous solution, and the luminescence intensity versus Fe^3+^ concentration plot. Inset: Stern–Volmer plot of Tb^3+^‐Alg‐TCPP quenched by Fe(NO_3_)_3_ aqueous solution. c,d) Comparison of the dominated emission peaks (λ_em_ = 544 nm) of Tb^3+^‐Alg‐TCPP after exposure to various aqueous solution with pH values from 10.0 to 1.0.

Cui et al. has proposed the possible underlying mechanism for the luminescence quenching in response to Fe^3+^ ions in the previous report.^31^ In our case, after Tb^3+^‐Alg‐TCPP hydrogels were treated with Fe^3+^ ions, there exists competitive absorption of excitation energy between Fe^3+^ and Tb^3+^ ions because Fe^3+^ ions and Tb^3+^ ions have a similar charge number and chelating capability. In order to investigate the possible leaching of Tb^3+^ after quenching with Fe^3+^, the quenching solution were evaporated and the residue were analyzed by EDX. It is noted that there is only very small amount of Tb element in the residue, which showed that the replacement degree of Tb^3+^ by Fe^3+^ is very low (Figure S5 and Table S2, Supporting Information). We proposed that Fe^3+^ can chelate with the uncoordinated carboxylic group of TCPP ligands, and the essential energy transfer may be shielded by Fe^3+^ ions and thus the luminescence of the Tb^3+^‐Alg‐TCPP hydrogels was quenched (Figure S6, Supporting Information).

Determination of the pH value is one of the most often performed measurements in modern laboratories. Among many different pH sensors, fluorescent‐based analyses are promising due to its low cost, convenient operations, high sensitivity, and temporal resolution.^32^, ^33^ Although much work about pH sensor has been reported,^34^ it also remains challenging to design and synthesize novel fluorescent pH sensors with an expanded pH sensing range.^35^, ^36^ The Tb^3+^‐Alg‐TCPP hydrogels used as pH sensors possess a series of desirable features including wide pH sensing range, good biocompatibility, and quick response, which could be better applied in biomedical applications and drug delivery in the future. Furthermore, when pH value decreased from 10.0 to 1.0, the perceptible photoluminescence changes could be detected by naked eyes under ultraviolet lamps (Figure 4c,d). The pH‐sensing mechanism of the Tb^3+^‐Alg‐TCPP hydrogels treated with increasing HCl concentration may be due to the protonation of carboxylate groups, which affects the coordination to Tb^3+^ ions and results in luminescence intensity decrease.^37^ Upon treatment with HCl solution, the H^+^ proton could destroy the covalent bond between TCPP and Tb^3+^ ions and impair the sensitization of the “antenna effect”.^38^ With the increasing concentration of H^+^ the luminescence intensity of the hydrogels was lessened and quenched eventually. It is also found that with the decreasing concentration of H^+^ again (pH = 1–10), the hydrogels showed some reversible pH sensing properties (Figure S7, Supporting Information).

Further study of time scale of quenching by Fe^3+^/H^+^ has also been performed. The lowest concentration of Fe^3+^ is 0.001 m, the time scale is 0.25, 0.5, 1, 12 h. After 0.5 h immersion in Fe^3+^, the luminescence intensity showed no obvious decreasing compared with 0.25 h immersion. This may be due to the fast diffusion of Fe^3+^ in the hydrogels, which are highly porous (Figure S8, Supporting Information). For H^+^ quenching, since at pH = 1, the quenching is almost complete, so pH = 3 is chosen. The time scale is 0.25, 0.5, 1, 12 h. The similar trend was observed, which may also be explained by the fast diffusion of H^+^ in the hydrogels (Figure S9, Supporting Information).

It is noted that Fe^3+^ will lower the pH of water. Then the corresponding control study was also performed. When the concentration of Fe^3+^ ion is 1 × 10^−3^ m, the pH is about 3.4. The results showed that the quenching is mainly due to Fe^3+^, but it must be recognized that at pH = 3.4, H^+^ can also quench the luminescence, but by a much less degree (Figure S10, Supporting Information).

Tb^3+^‐Alg‐TCPP hydrogels are considered to be biocompatible since alginate matrix is an environmentally friendly natural biomacromolecule. To further confirm the cytotoxicity of Tb^3+^‐Alg‐TCPP hydrogel materials, 3‐(4,5‐dimethylthiazol‐2‐yl)‐2,5‐diphenyltetrazolium bromide (MTT) was performed to assay cell viability.^39^ The viability of rat aortic endothelial cells (RAECs) of all the groups after being exposed to different concentration of Tb^3+^‐Alg‐TCPP aqueous dispersion exceeds 95% except that at the highest concentration of Tb^3+^‐Alg‐TCPP aqueous dispersion (1:2) the biological activity was 80% (**Figure** **5**). The MTT results confirmed that Tb^3+^‐Alg‐TCPP hydrogels had almost no toxicity, showing good biocompatibility. Therefore, we hope Tb^3+^‐Alg‐TCPP hydrogels would bring new possibilities for the use and design of luminescent hybrid biomaterials in biomedical applications.

**Figure 5:**
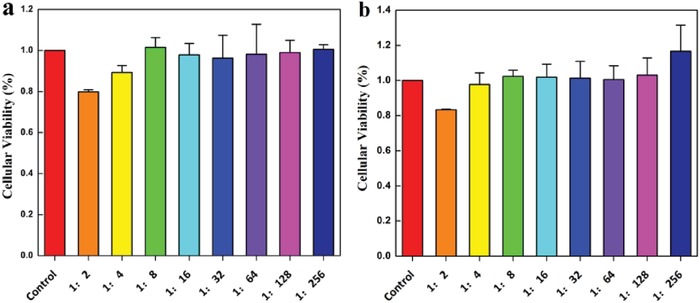
The comparison of cellular viability assayed by MTT of a) Alg and b) Tb^3+^‐Alg‐TCPP. The RAECs were incubated for 24 h before evaluation.

In summary, our work proposes a facile new method to fabricate highly luminescent hydrogels with covalently bonded lanthanide complex to alginate biopolymer matrix. The proposed strategy demonstrates a rapid, economical, and green pathway to prepare novel hybrid materials with excellent biocompatibility. This new type of alginate hydrogels can be used as bifunctional sensors. The luminescent hydrogels show relatively high selectivity and sensitivity for detecting Fe^3+^ in aqueous solution. They are also sensitive to pH with wide pH sensing range and high sensitivity. This work may provide a facile route of designing multifunctional hybrid luminescent materials to be utilized in the field of fluorescent sensors.

## Conflict of Interest

The authors declare no conflict of interest.

## Supporting information

Supplementary
