# Supplementary material for: Covalent Grafting Terbium Complex to Alginate Hydrogels and Their Application in Fe3+ and pH Sensing
Source: Glob Chall. 2018 Nov 13;3(2):1800067. doi: 10.1002/gch2.201800067 (PMC6607234; doi:10.1002/gch2.201800067)
Supplement: Supplementary file 1 — Supplementary [file GCH2-3-na-s001.pdf]

Copyright WILEY-VCH Verlag GmbH & Co. KGaA, 69469 Weinheim, Germany, 2018.

Global Challenges
Open Access

Supporting Information

for *Global Challenges,* DOI: 10.1002/gch2.201800067

Covalent Grafting Terbium Complex to Alginate Hydrogels and Their Application in Fe^{3+} and pH Sensing

Zeyu Zhang, Fengyi Liu, Quanqing Xu, Han Zhu, Aixin Zhu, and Junfeng Kou**

Copyright WILEY-VCH Verlag GmbH & Co. KGaA, 69469 Weinheim, Germany, 2016.

Supporting Information

Covalent grafting terbium complex to alginate hydrogels and their application in Fe^{3+} and pH sensing

Ze-yu Zhang, Feng-yi Liu*, Quan-qing Xu, Han Zhu, Ai-xin Zhu and Jun-feng Kou*

1. Experimental section

1.1 Materials

Terbium(III) chloride hexahydrate ($TbCl_3 \cdot 6H_2O$, 99%) was purchased from Aladdin Industrial Corporation, Shanghai, China. Alginate was supplied by J&K Scientific Corporation, Beijing, China. meso-tetra(4-carboxy-phenyl)porphine (TCPP) was supplied by Frontier Scientific Inc, UT, USA. Hydrochloric acid (HCl) and absolute alcohol used were of analytical grade, and used without further purification. Deionized water was used throughout the experiments.

1.2. Preparation of Tb^{3+}-Alg-TCPP hydrogel and aerogel

The hydrogel and aerogel materials in this work were obtained according to the previously reported procedure.[1]

1.2.1. Hydrogel

A 2 % (w/w) solution of alginates dissolved in deionized water, which was added dropwise to a $TbCl_3$ solution (0.1 M; 100 ml) under stirring at room temperature. Gel microspheres were matured in the Tb^{3+} solution for 24 h, and then the gel microspheres was separated from the cationic solution and washed with deionized water. TCPP was dissolved into sodium hydroxide aqueous solution and adjusted pH value of the solution to 10. Tb^{3+}-Alg hydrogels was soaked in the saline solution of TCPP, and stirred with magnetic stirring apparatus for twelve hours at room temperature. The microspheres were separated from the cationic

solution and washed with plenty of deionized water, then obtained colorless and transparent hydrogel microspheres (Tb^{3+}-Alg-TCPP) with superior photoluminescence character.

1.2.2. Aerogels

The Tb^{3+}-Alg-TCPP hydrogel microspheres were successively immersed in a series of ethanol-water baths with increasing alcohol concentration (10, 20, 30, 40, 50, 60, 70, 80, 90, and 100%) for 1 h for each solution. Finally, the hydrogels were gradually changed into alcogels. These alcogel microspheres were dried by a Polaron 3100 apparatus under supercritical CO_2 conditions (74 bar, 31.5°C) to obtain Tb^{3+}-Alg-TCPP aerogels.

1.3. Experimental details for luminescent sensing of metal ions

Tb^{3+}-Alg-TCPP hydrogels were immersed in different aqueous solutions of various metal ions including K^+, Ca^{2+}, Mg^{2+}, Cd^{2+}, Al^{3+}, Zn^{2+}, Ag^+, Ni^{2+}, Mn^{2+}, Co^{2+} and Fe^{3+} (1 mM), and then dealt with fluorescent response testing. Quenching by the mixture of metal ions was also performed in the similar method. To better understanding the fluorescent response of Tb^{3+}-Alg-TCPP hydrogels towards Fe^{3+} cations, fluorometric titration was adopted with the addition of different concentration of $Fe(NO_3)_3$ solution to Tb^{3+}-Alg-TCPP hydrogels.

1.4. Experimental details for luminescent sensing of pH

The Tb^{3+}-Alg-TCPP hydrogels were immersed in a series of aqueous solutions with different pH value from 10.0 to 1.0, respectively. The photoluminescence spectra of the processed samples were obtained by the Hitachi F-7000 fluorescence spectrophotometer with a xenon lamp as an excitation source.

2. Characterizations

Scanning electron micrographs and chemical composition (elemental maps) of the aerogel microspheres were obtained using a NOVA NANOSEM-450 apparatus after gold metallization. Thermogravimetric analysis was measured using a STA449F31 apparatus under N_2 atmosphere (20 mL min^{-1}, 25-800 °C, 5 °C min^{-1}) with 10 mg sample.

2.1. Nitrogen adsorption/desorption isotherms

Nitrogen adsorption/desorption isotherms were performed on a JWGB-BK132F analyzer at 77 K after outgassing the sample at 323 K under a vacuum until a stable 3 × 10^{-3} Torr pressure was obtained without pumping.

2.2. Biocompatibility assessment

To assess the biocompatibility of the Tb^{3+}-Alg-TCPP, RAECs (rat aortic endothelial cells) were grown in medium supplemented with the prepared powder of aerogels. The cell viability was measured by MTT viability assay with a colorimetric measure of detecting the mitochondrial activity. Cells were cultured in a 96-well plate (Costar, approximately 4×10^3 cells per well) with DMEM Medium containing 10% FBS and different concentrations of the power dispersed in deionized water for 24 h. Then, 20 μl of MTT solution (5 mg/ml MTT in phosphate buffer solution, pH 7.4) was added to each plate and incubated for 4 h at 37℃. After removing the medium, intracellular formazan crystals were extracted into 150 μl of DMSO and quantified by measuring the absorbance of the cell lysate at 490 nm.

2.3. Thermogravimetric analysis

The curves of thermogravimetric analysis (TGA) and their differential thermogravimetric analysis (DTG) of the aerogel samples are presented in Figure S11. The weight loss at the temperature range of 20-190℃ can be attributed to the loss of surface and inner moisture by evaporation. The stage of mass loss between the range of 190-360℃ can be ascribed to the intensive evolution of small molecules and the degradation of alginate with the fracture of glycosidic bonds to form intermediate compounds, releasing small molecules such as H_2O and CO_2.[2] The curves of thermogravimetric analysis of the aerogel samples shows that the Tb^{3+}-Alg-TCPP has considerable thermal stability up to 210℃.

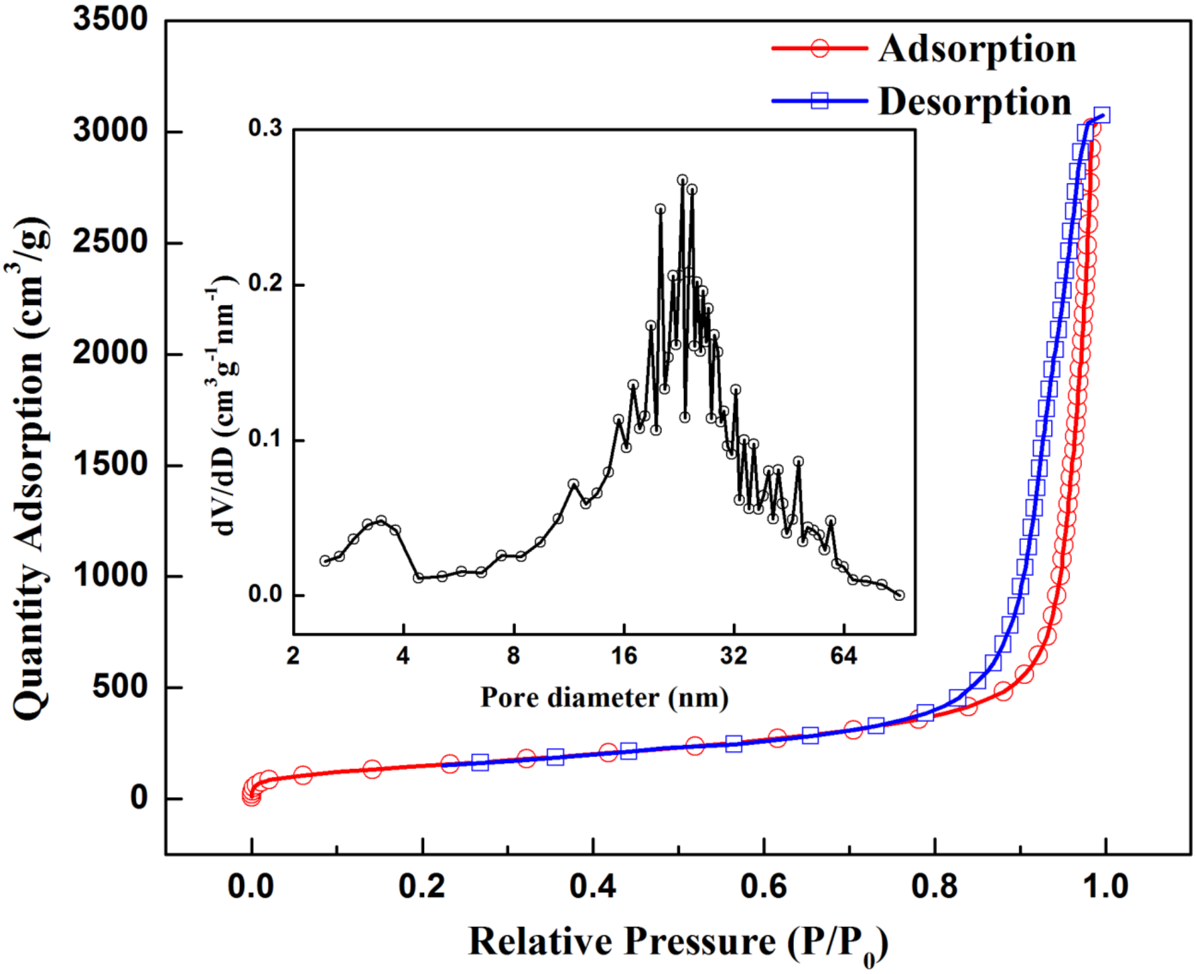

Figure S1. Nitrogen adsorption-desorption isotherms of Tb^{3+}-Alg-TCPP aerogel.

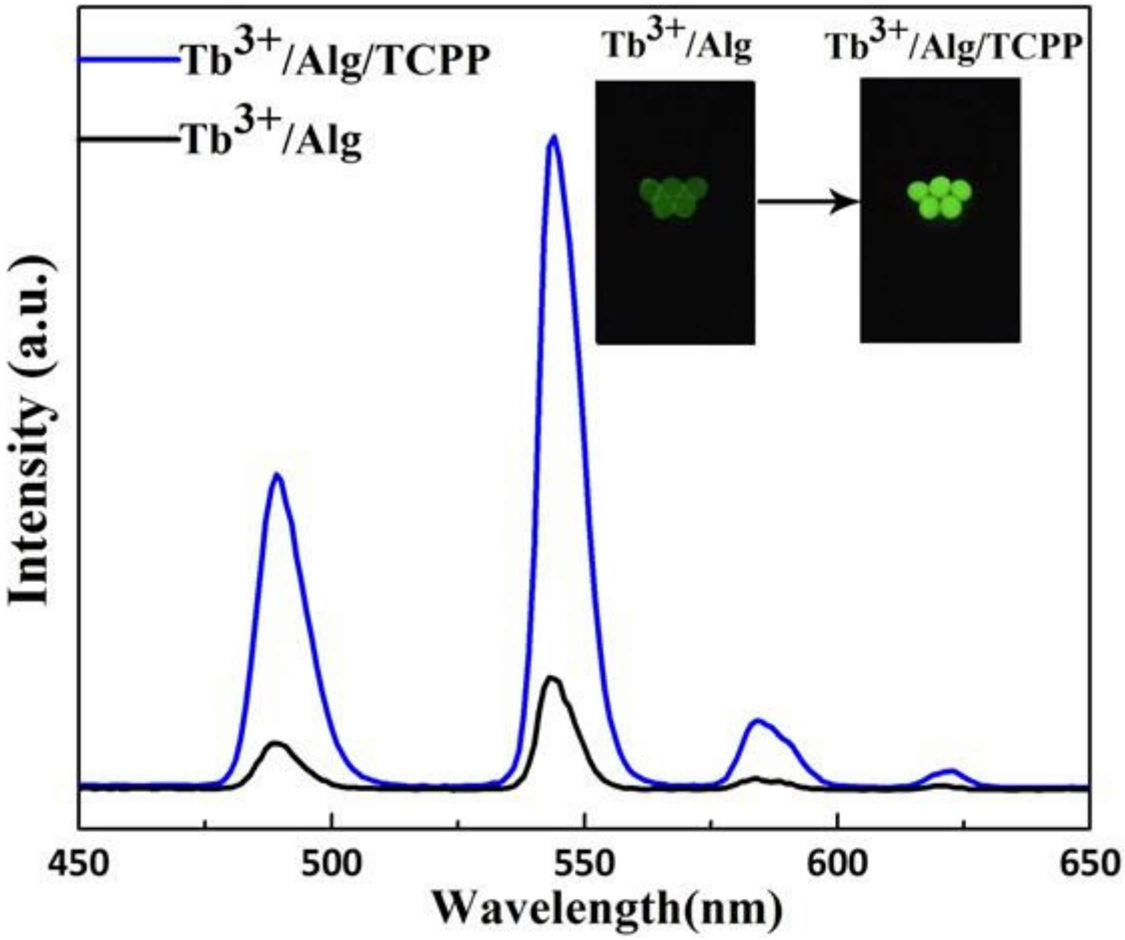

Figure S2. The comparison of the emission spectra of Tb^{3+}-Alg and Tb^{3+}-Alg-TCPP. Inset shows the photograph of hydrogels under the irradiation of UV light.

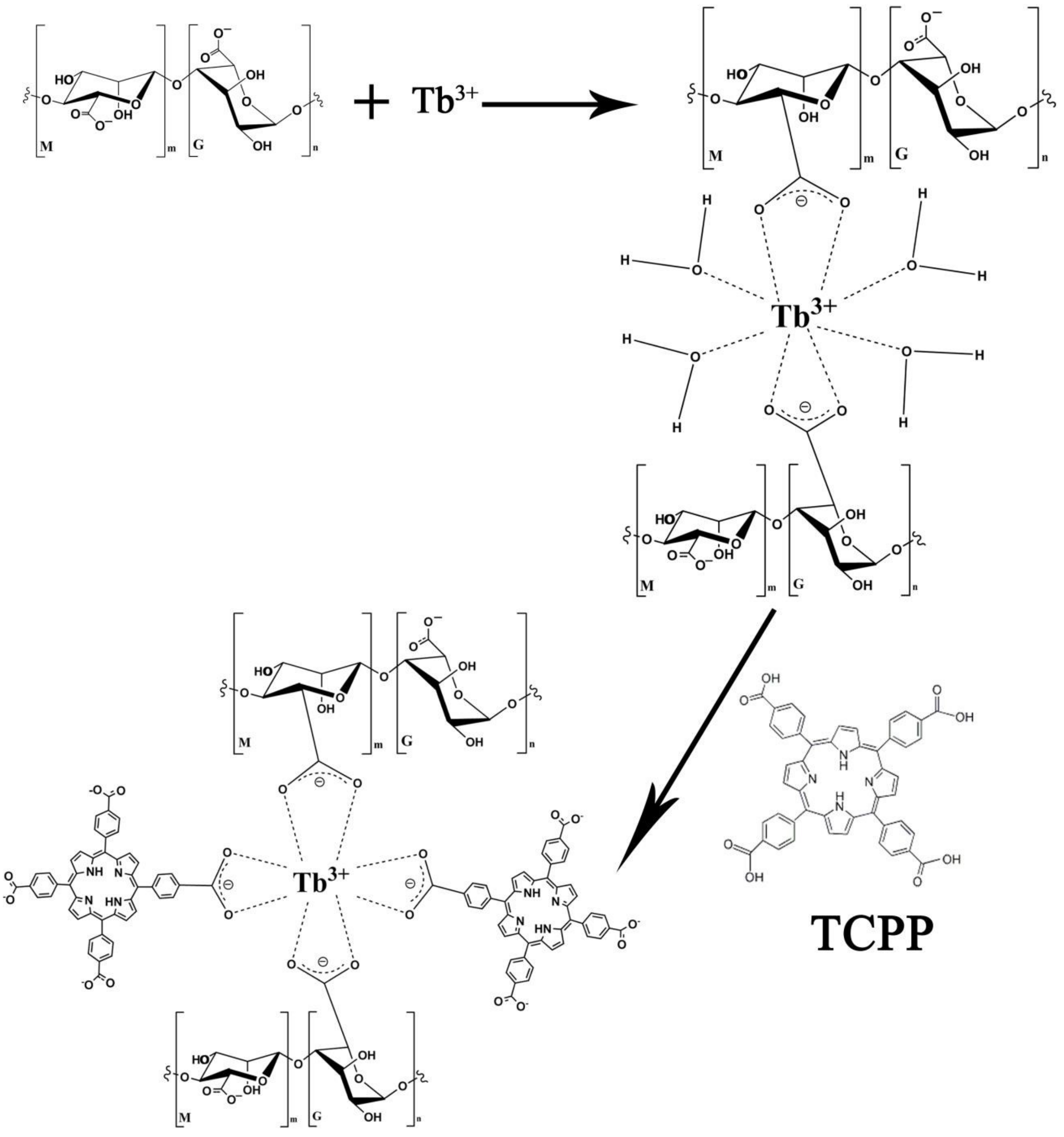

Figure S3. The schematic diagram of the linkage between Tb^{3+} and alginate, TCPP.

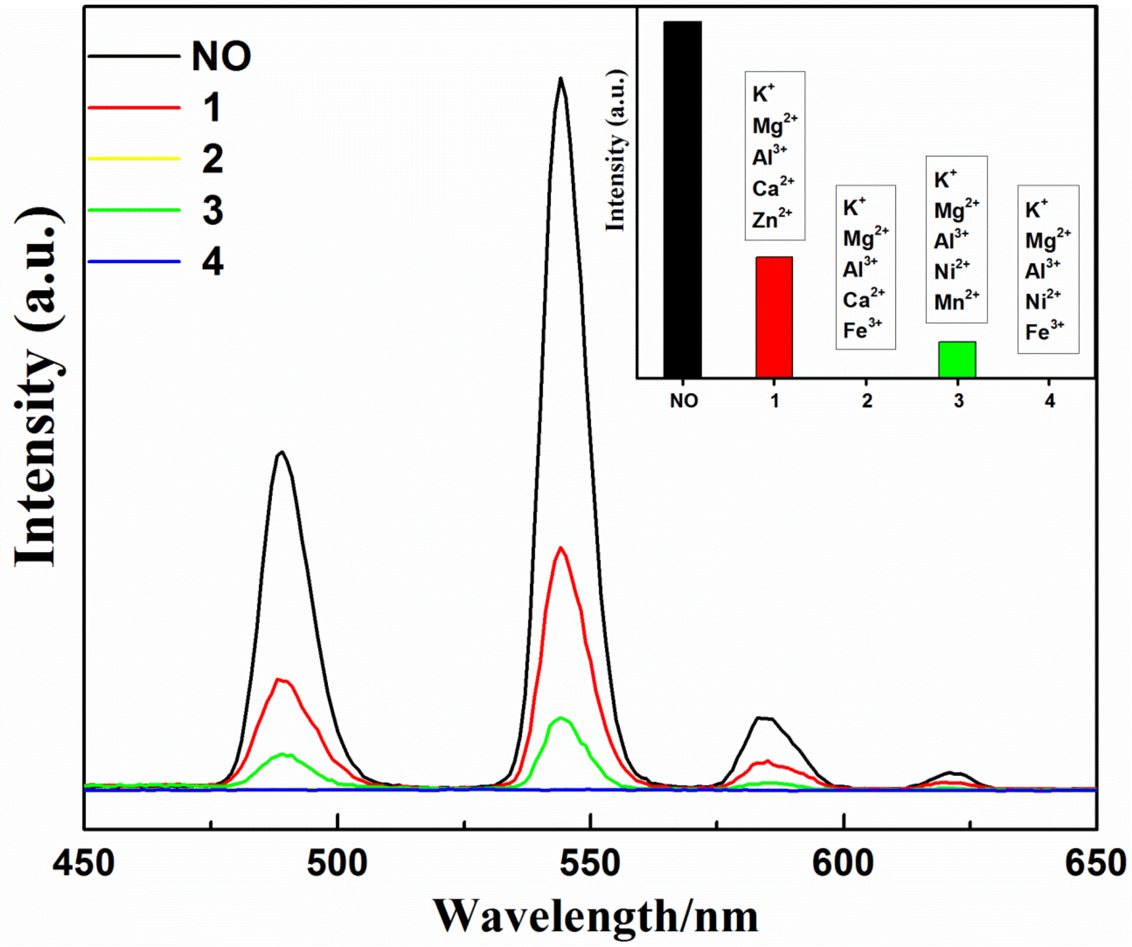

Figure S4. The comparison of the emission spectra of Tb^{3+}-Alg-TCPP immersed in different mixed metal ions solution with the same concentration (1 mM). Inset shows the fluorescence intensity (λ_{em} = 544 nm) with different mixed metal ions. "NO" denote the fluorescence intensity of the Tb^{3+}-Alg-TCPP hydrogels without treatment by metal ions.

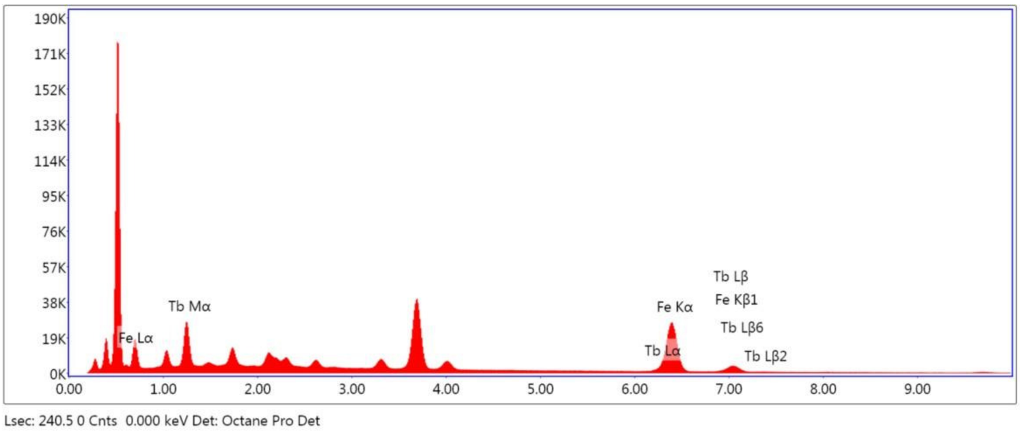

Figure S5. The EDX-analysis for the residue of the Fe^{3+} solution of 1 mM after being soaked with the Tb^{3+}-Alg-TCPP hydrogels.

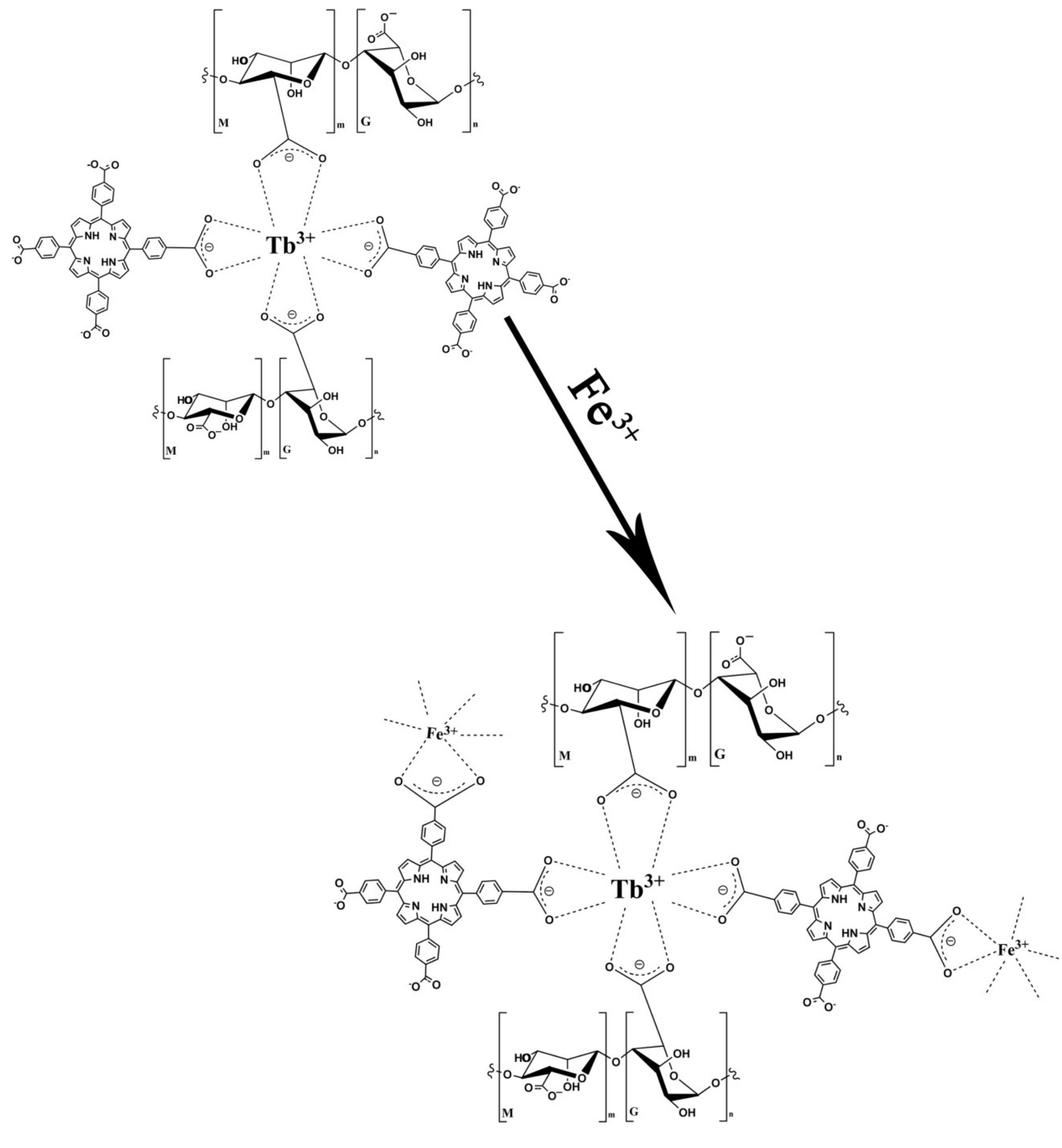

Figure S6. Proposed quenching mechanism of the Tb^{3+}-Alg-TCPP hydrogels by Fe^{3+} solution.

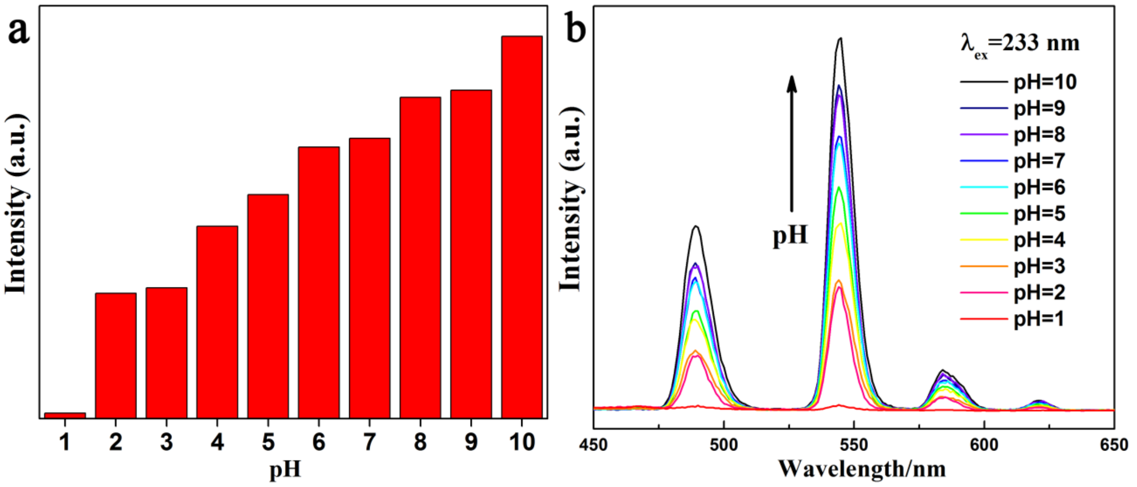

Figure S7. The fluorescence intensity (λ_{em} = 544 nm) with the treatment of pH from 1to 10. (a). The comparison of the emission spectra of Tb^{3+}-Alg-TCPP with the treatment of pH from 1to 10.

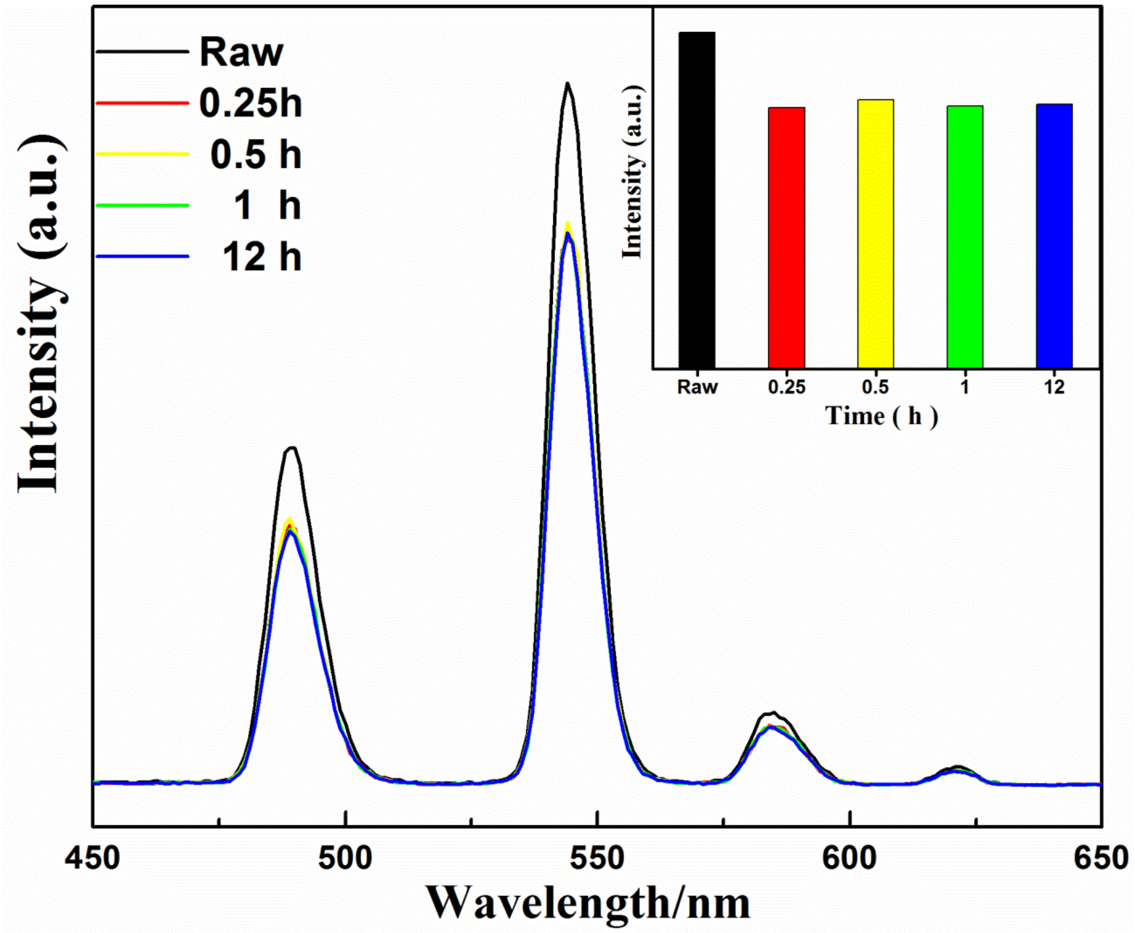

Figure S8. The comparison of the emission spectra of Tb^{3+}-Alg-TCPP with different immersion time with Fe^{3+} solution of 0.001 mM. Raw denotes Tb^{3+}-Alg-TCPP were not dealt with Fe^{3+}. Inset shows the fluorescence intensity (λ_{em} = 544 nm) with different immersion time.

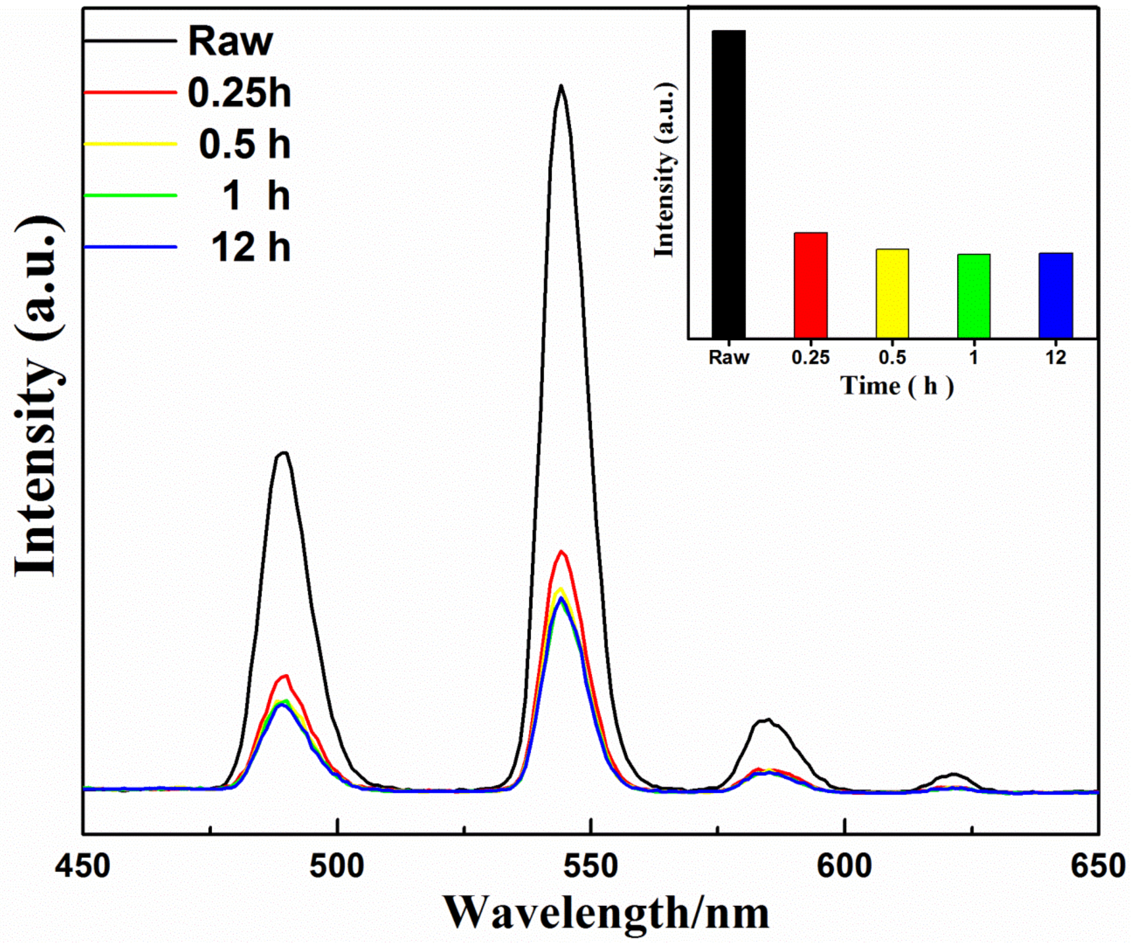

Figure S9. The comparison of the emission spectra of Tb^{3+}-Alg-TCPP with different immersion time at pH= 3. Raw denotes the Tb^{3+}-Alg-TCPP hydrogels immersed in the deionized water with pH= 7. Inset shows the fluorescence intensity (λ_{em} = 544 nm) with different immersion time.

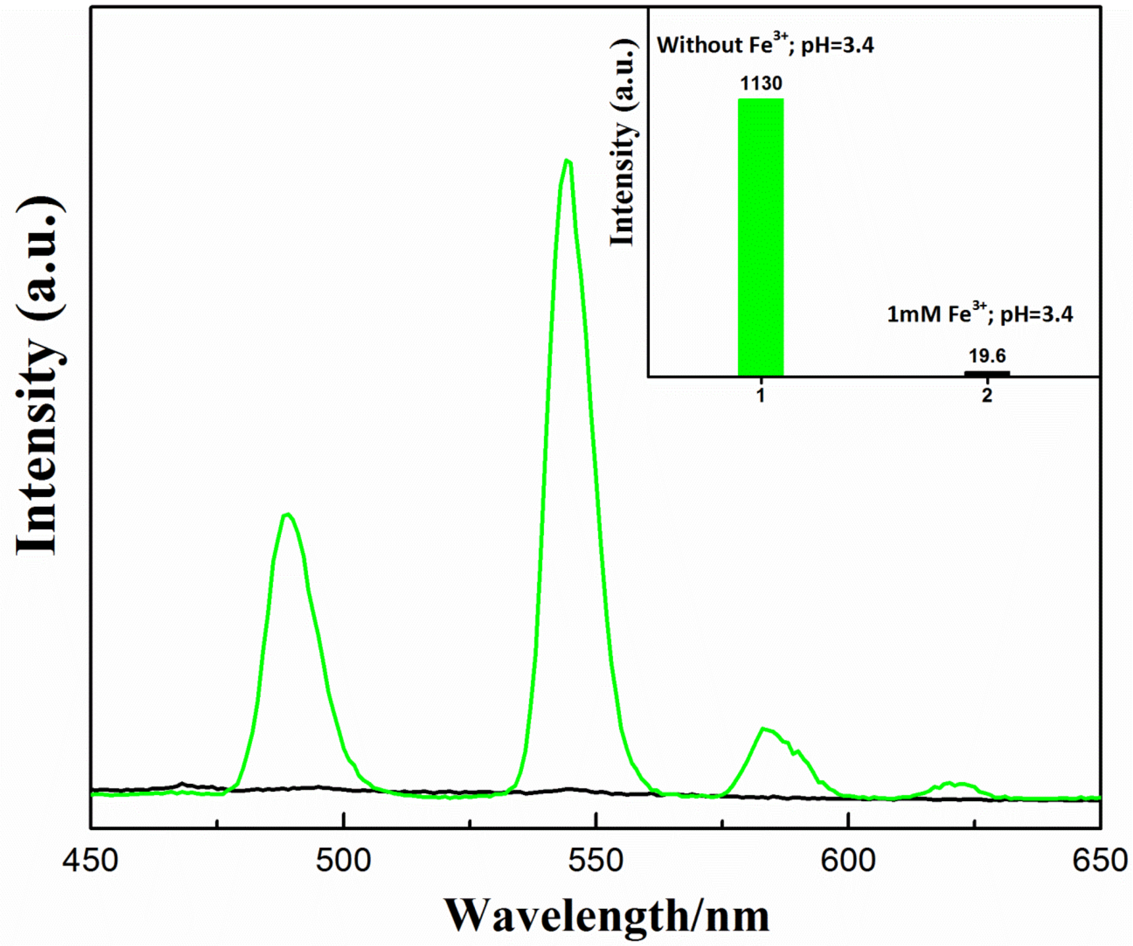

Figure S10. The comparison of the emission spectra of Tb^{3+}-Alg-TCPP immersed in 1mM Fe^{3+} (pH= 3.4) and H^{+} solution (pH= 3.4) without Fe^{3+}. Inset shows the fluorescence intensity (λ_{em} = 544 nm) with Fe^{3+} or without.

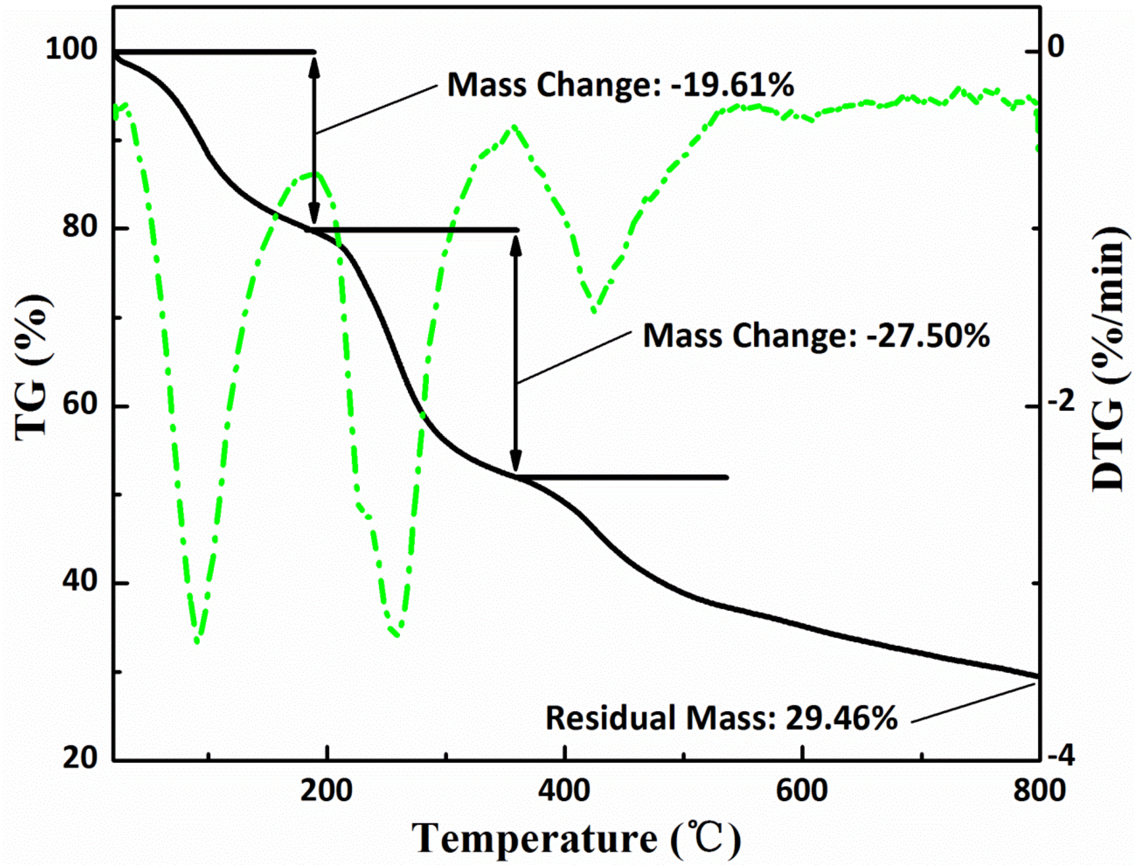

Figure S11. The TG and DTG curves of Tb^{3+}-Alg-TCPP.

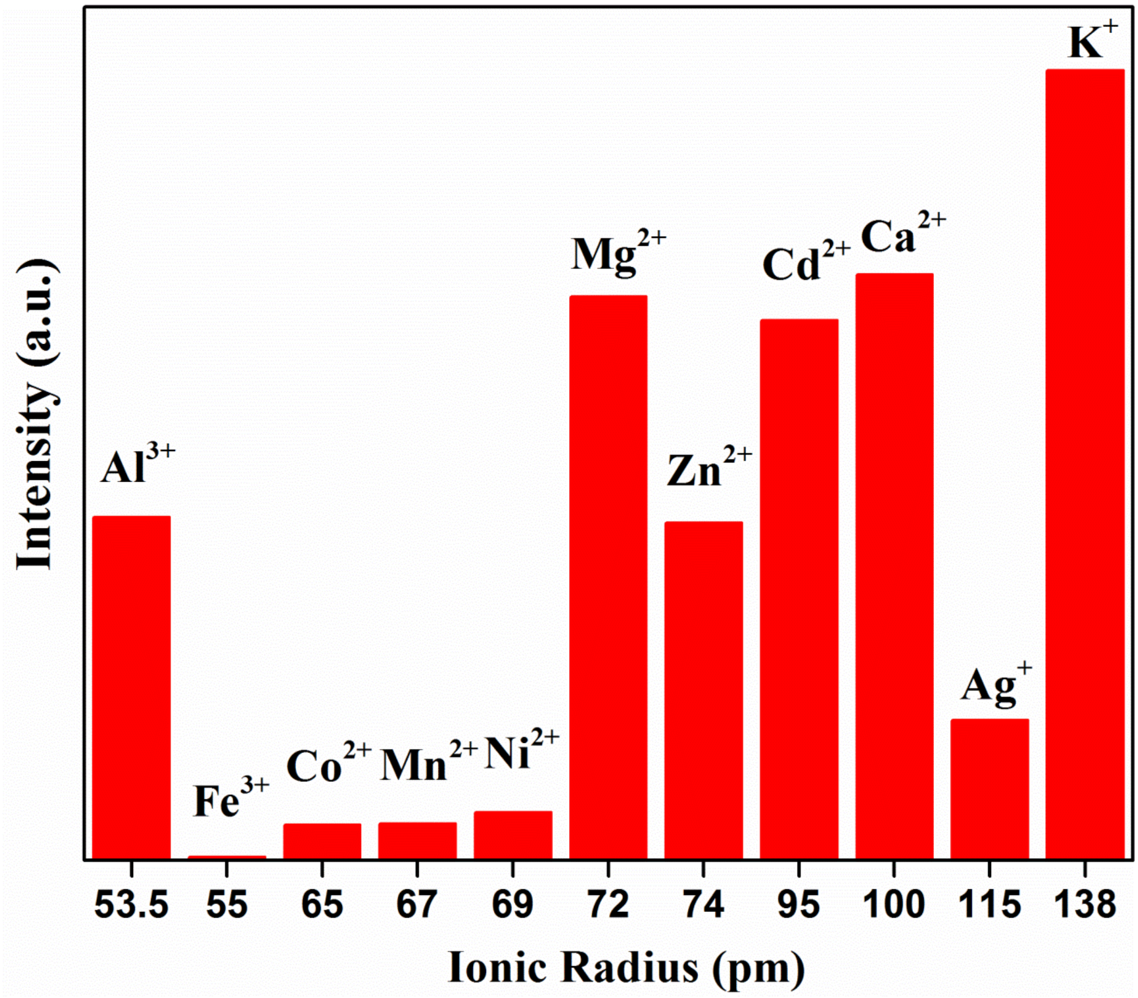

Figure S12. After treated by different metal ions (1 mM), the relationship between fluorescence intensity (λ_{em} = 544 nm) of Tb^{3+}-Alg-TCPP hydrogels with ionic radius. (Ionic radius data are from W. M. Haynes. CRC Handbook of Chemistry and Physics. 93rd ed. Boca Raton: CRC press Inc, 2012).

Table S1. The percentage of element of the Tb^{3+}-Alg-TCPP Aerogels in the EDX-analysis.

Element	Weight %	Atomic %	Error %
C	22.18	39.38	7.55
N	0.33	0.50	20.31
O	32.16	42.86	7.65
Tb	31.23	4.19	3.05

Table S2. The percentage of element of the residue of the Fe^{3+} solution after being soaked with Tb^{3+}-Alg-TCPP hydrogels in the EDX-analysis.

Element	Weight %	Atomic %	Error %
O	29.56	55.90	7.77
Tb	4.59	0.87	10.73
Fe	44.50	24.11	2.50
